# Tissue regulatory T cells

**DOI:** 10.1111/imm.13208

**Published:** 2020-06-24

**Authors:** Prudence PokWai Lui, Inchul Cho, Niwa Ali

**Affiliations:** ^1^ Centre for Stem Cells and Regenerative Medicine School of Basic and Biomedical Sciences King's College London London UK; ^2^ The Francis Crick Institute London UK

**Keywords:** inflammation, regulatory T cells, tissue regulatory T cells, tissue repair

## Abstract

Foxp3^+^ CD4^+^ regulatory T cells (Tregs) are an immune cell lineage endowed with immunosuppressive functionality in a wide array of contexts, including both anti‐pathogenic and anti‐self responses. In the past decades, our understanding of the functional diversity of circulating or lymphoid Tregs has grown exponentially. Only recently, the importance of Tregs residing within non‐lymphoid tissues, such as visceral adipose tissue, muscle, skin and intestine, has been recognized. Not only are Tregs critical for influencing the kinetics and strength of immune responses, but the regulation of non‐immune or parenchymal cells, also fall within the purview of tissue‐resident or infiltrating Tregs. This review focuses on providing a systematic and comprehensive comparison of the molecular maintenance, local adaptation and functional specializations of Treg populations operating within different tissues.

## Graphical abstract

Foxp3^+^ CD4^+^ regulatory T cells (Tregs) are an immune cell lineage endowed with immunosuppressive functionality. Only recently, the importance of Tregs residing within non‐lymphoid tissues, such as visceral adipose tissue, muscle, skin and intestine, has been recognized. Not only are Tregs critical for influencing the kinetics and strength of immune responses, but the regulation of non‐immune or parenchymal cells, also falls within the purview of tissue‐resident or infiltrating Tregs.

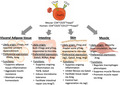

**Abbreviations**
Areg: amphiregulin
CAR‐Tregs: chimeric antigen receptor Tregs
DTR: diphtheria toxin receptor
EGFR: epidermal growth factor receptor
Foxp3: Forkhead box P3 transcription factor
HF: hair follicle
HFSC: hair follicle stem cell
LN: lymph node
MHC: major histocompatibility complex
PPAR*γ*: peroxisome proliferator‐activated receptor ; *γ*
pTregs: peripherally derived Tregs
TCR: T‐cell receptor
Teffs: CD4^+^ T effector cells
TGF‐*β*: transforming growth factor ; *β*
Tregs: regulatory T cells
tTregs: thymic‐derived Tregs
VAT: visceral adipose tissue

## Introduction

The concept of T cells suppressing the immune system began in the early 1970s.^1^, ^2^, ^3^ However, the field suffered scrutiny from the early 1980s to the 1990s, due to the lack of unique phenotypic markers and molecular mechanisms underlying immunosuppression (reviewed in refs 4, 5). Such a narrative began to change when Sakaguchi and colleagues identified a subset of thymic CD4^+^ T cells in murine lymph nodes (LN) expressing high levels of interleukin‐2 receptor‐*α* (IL‐2R*α*; CD25), capable of suppressing autoimmunity.^6^ Shortly after, Forkhead/winged‐helix transcription factor 3 (Foxp3) was recognized as a critical phenotypic and functional regulatory T cell (Treg) marker, which is mutated in ‘scurfy’ mice,^7^ and also in human immunodysregulation polyendocrinopathy enteropathy X‐linked patients.^8^ Foxp3 mutation in both scenarios results in an accelerated and fulminant systemic autoimmunity. These major breakthroughs led to the rebranding from ‘suppressor’ T cells to the present ‘regulatory’ T cells (i.e. Tregs), and the exponential bloom of our understanding in Treg development and functions (reviewed in ref. 9). We have recently come to appreciate populations of Tregs residing within non‐lymphoid tissues, collectively termed ‘tissue Tregs’. Here, we provide a comparison of how Tregs residing in tissue [such as visceral adipose tissue (VAT), intestine, skin or muscles] are specialized for orchestrating tissue homeostasis as regulators of both immune and non‐immune cells.

## General definition and heterogeneity of Tregs

Besides CD25, Tregs may express other activation markers, such as co‐stimulatory/co‐inhibitory molecules [CTLA‐4, inducible T‐cell costimulator (ICOS), CD27, LAG‐3, LAP, CD69, PD‐1), tumour necrosis factor receptor superfamily (TNFRSF) members (OX40, GITR), cell adhesion‐related markers (CD49b, CD62L) and migratory receptors guiding to peripheral destinations (CD103, CCR4, CCR5, CCR6, CCR7).^10^ In particular, Foxp3 is exclusively expressed in CD4^+^ CD25^+^ murine Treg populations, but at low levels in CD4^+^ CD25^−^ effector T cells (Teffs) and largely absent in CD8^+^ T cells.^7^, ^11^ Importantly, Foxp3 is indispensable for Treg lineage development and immunosuppressive functions.^7^, ^11^, ^12^, ^13^ Hence, the general consensus is that the murine Treg lineage is defined as CD4^+^ CD25^high^Foxp3^+^. Yet, in human peripheral blood, not all Foxp3‐expressing cells correlate with high levels of CD25 expression (reviewed in ref. 10), nor immunosuppressive function.^14^ The IL‐7R*α* chain, CD127, is inversely correlated with Foxp3 expression, and CD4^+^ CD127^low^ T cells in humans display similar suppressive capacity to CD4^+^ CD25^high^ T cells *in vitro*.^15^ Hence, human Tregs are more accurately defined as CD4^+^ CD25^high^ CD127^low/−^ Foxp3^+^.

While CD4^+^ CD25^high^ CD127^low/−^ Foxp3^+^ is the phenotypic backbone of Tregs, multiple flow cytometry‐based and RNA sequencing studies indicate that both mouse and human Tregs are highly heterogeneous (reviewed in ref. 16). One subdivision is based on Treg origin. CD25^high^ CD4^+^ thymocytes initially undergo a positive selection process and develop into Treg precursors. This is triggered through T‐cell receptor (TCR) engagement with high‐affinity tissue‐specific self‐peptides on major histocompatibility complex type II (MHCII) presented by antigen‐presenting cells and medullary thymic epithelial cells. Stimulated by the cytokines IL‐2 or IL‐15, and in combination with transforming growth factor*‐β* (TGF‐*β*) signalling, Treg precursors differentiate into fully committed thymic Tregs (tTregs).^17^, ^18^ Peripherally derived Tregs (pTregs) can be generated from naive Foxp3^−^ CD4^+^ T cells in secondary lymphoid organs and peripheral tissues, in response to various cytokines upon antigen exposure, including TGF‐*β*.^19^ Furthermore, Tregs may also be subdivided according to their differentiation state (CD45RA^+^ naive, nTregs, or CD45RA^−^ activated, aTregs), and their degree of activation (CD44^low^ CD62L^high^ quiescent central, cTregs, or CD44^high^CD62L^low^ effector, eTregs) and by their expression of core T helper (Th) lineage‐defining transcription factors (T‐bet for Th1, Gata3 for Th2, and RoR*γ*t for Th17) (reviewed in refs 16, 20).

## Phenotypic dynamics of tissue Tregs

Unsurprisingly, murine Tregs share the core phenotypic backbone of their circulatory and lymphoid counterparts, regardless of their tissue of residence. For example, 63% of the VAT Treg transcriptome overlaps with that of LN Tregs, including the overexpression of hallmark activation markers, including *Cd25*,* Gitr*,* Ctla4*,* Ox40* and *Klrg1*.^21^ Indeed, genome‐wide chromatin accessibility profiling (ATAC‐seq) has indicated that VAT, muscle and colon Tregs share 79% of their open chromatin regions with their splenic counterparts, whereas only 3% are unique to these ‘pan‐tissue’ Tregs.^22^ Re‐analysis of existing microarray data revealed that of ~2000 tissue Treg‐associated genes, 9% are shared among VAT, muscle and colon, including up‐regulation of the IL‐33 receptor (*Ilrl1*, also known as ST2) and amphiregulin (*Areg*) genes.^22^ Another example is the peroxisome proliferator‐activated receptor *γ* gene (*Pparγ*), expressed in Tregs residing in VAT, muscle and neonatal liver.^22^, ^23^ Another single‐cell RNA sequence profiling also demonstrated the resemblance between ear skin Tregs and a subset of colon Tregs, sharing expression of *Areg*,* Ilrl1* and *Il10*, but also *Gata3*,* Rora* and *Tnfrsf4*.^24^ Additionally, inhibitors of DNA binding 3 (Id3) is also consistently absent among Tregs in skin, fat, colon and lung, but is abundant in circulating or lymphoid Tregs.^25^

Human Tregs share <10% of overall mRNA expression with their mouse counterparts, such as *Rora* and *Tnfrsf18* in skin, and *Ikzf3* in colon.^24^ Contrarily, multiple mRNA signatures, such as serine/threonine kinase (PIM1/2), cAMP‐related genes (PDE3B/4B/4D) or G‐protein signalling regulator (RGS1/2), are opposingly expressed between human and mouse tissue Tregs.^24^ Whether this directly reflects species differences in function remains an open question. It may be reasonable to characterize tissue Tregs based on these commonly shared ‘pan‐tissue’ phenotypes, at least in mouse. Despite their phenotypic resemblance, certain genes remain uniquely expressed in each murine tissue Treg, which we will explore below.

## Conventional anti‐inflammatory functions of Tregs

As its name suggests, Tregs regulate and suppress a variety of immune cell types (such as macrophages, dendritic cells, CD4^+^ Teffs and CD8^+^ T cells) (reviewed in ref. 26). Three general modes of suppression have been proposed: (i) cell‐to‐cell contact via Treg–cytotoxic T‐lymphocyte antigen 4 (CTLA‐4) regulation of CD28 co‐stimulation, an essential secondary signal for T‐cell activation. Mechanistically, CTLA‐4 physically removes and down‐regulates the ligands CD80/CD86 on target cells, through a process termed trans‐endocytosis;^27^ (ii) Treg production of cytokines (IL‐10 and TGF‐*β*); and (iii) metabolic alteration, whereby CD25^hi^ Tregs consume the T‐cell growth factor IL‐2, and restrict expansion of other T cells (reviewed in ref. 28).

The initial response towards tissue damage or infection is an acute inflammation. Upon removal of pathogens or damaged cells, Tregs dampen inflammation by secreting anti‐inflammatory cytokines, and regulating the recruitment/activation of other immune cells (reviewed in ref. 29). For example, Tregs directly suppress neutrophil recruitment via IL‐10, but also indirectly induce neutrophil apoptosis via TGF‐*β*. Hence, TGF‐*β* signalling not only functions as an inducer of the Treg lineage, but also as a facilitator of Treg‐mediated immunosuppression. Additionally, Tregs also promote macrophage polarization from a more inflammatory to anti‐inflammatory phenotype. Perhaps the best‐established suppressive function of Tregs is the regulation of pro‐inflammatory cytokines interferon‐*γ* (IFN‐*γ*) and tumour necrosis factor‐*α* (TNF‐*α*), as well as the abundance and activation of CD4^+^ and CD8^+^ T cells.

The major criterion for successful wound healing is the initiation of tissue remodelling to restore tissue integrity (reviewed in ref. 30). However, through inability to clear pathogens and/or dysregulation of the inflammatory process itself, tissues may transition to a chronic inflammatory and fibrosis‐associated phase. Fibrosis is broadly characterized by excessive extracellular matrix deposition, and eventually, tissue scarring. Although type 2 cytokines and macrophage‐derived TGF‐*β* promote fibrosis, Treg‐derived TGF‐*β* and IL‐10 play an opposing role (reviewed in ref. 31). In particular, the link between inflammation and fibrosis is best exemplified in the neutrophil‐ and macrophage‐deficient PU.1 null mice. This mutant lacks the major components of an inflammatory response. Yet, cutaneous wound closure kinetics are equivalent to wild‐type animals, with minimal tissue scarring.^32^ It was later discovered that the knockdown of osteopontin (an inflammation‐dependent gene) improves collagen assembly, limits neutrophil, mast cell and macrophage recruitment, increases neovascularization, minimizes fibrosis and reduces scarring.^33^ Taken together, the suppression of inflammation may minimize tissue fibrosis. It is, therefore, logical that tissue Tregs also play an essential role in suppressing fibrosis and promoting tissue repair, which we will explore thoroughly in this review. Unless otherwise stated, the majority of the data discussed below are derived from murine studies. We review our current knowledge on the phenotypes, origin and functions of four well characterized non‐lymphoid Treg populations, which reside in VAT, intestine, skin and skeletal muscle.

## Visceral adipose tissue Tregs

Visceral adipose tissue refers to the white adipocytes localizing around various organs, which function primarily as reservoirs of energy storage (reviewed in ref. 34). In the steady‐state, murine VAT Tregs account for ~50% of CD4^+^ T cells.^21^ Remarkably, VAT Tregs uniquely express peroxisome proliferator‐activated receptor *γ* (PPAR*γ*),^35^ a transcription factor usually restricted to adipocytes to drive adipose tissue development. Conditional knockout of *PPARγ* in Tregs results in a ~70% reduction of VAT Treg abundance, and the down‐regulation of the VAT Treg‐associated transcripts *Ccr2*,* Gata3*,* Klrg1* and *Cd69* (an early activation and tissue residency marker).^35^, ^36^ These findings indicate that PPAR*γ* can act as a specific inducer and regulator of VAT Treg identity.

### Thymic origin of VAT Tregs

Several lines of evidence indicate that VAT Tregs are likely of thymic origin. First, >90% of VAT Tregs express high levels of the thymic‐associated markers Helios and Nrp‐1, at comparable levels to splenic and LN Tregs.^37^ Additionally, when pooled Teffs from 8‐week‐old CD45.1^+^ Foxp3^iGFP^ reporter mice are transferred into 20‐week‐old congenic CD45.2^+^ Foxp3^iGFP^ recipients, donor‐derived VAT Tregs are absent, suggesting that Teffs are unlikely to be the predominant source.^37^

To better understand the origin of tissue Tregs, TCR sequencing analysis may be performed. In brief, the majority of T cells, including Tregs, express highly diverse TCRs.^38^ Each TCR consists of a combination of *α* and *β* chains (*αβ* TCR), with each chain containing three complementary determining regions (CDR1–3).^39^ The CDR3 region of a TCR is often in direct contact with the antigen, and so plays a defining role in the interaction with the peptide–MHC complex. Hence, CDR3 diversity is often reflective of T‐cell specificity, and in turn their clonality. If two T cells express identical CDR3 sequences, then they are likely derived from a clonally expanded T cell. Shared TCR sequences between Tregs and Teffs suggest the two populations may recognize the same antigens. Under the assumption that TCR repertoire remains unaltered during the transition from naive Teffs to pTregs, TCR similarity indirectly indicates whether a Treg population originates from Teffs. In VAT, the Treg TCR repertoire (specifically CDR3*α* and CDR3*β* sequences) resembles that of LN Tregs, but is distinct from VAT or LN Teffs.^37^ The lack of CDR3 overlap between VAT Tregs and VAT Teffs indicates distinct antigen specificities. Thus, VAT Tregs are unlikely to originate from Teffs, suggesting minimal pTreg input.^37^

Interestingly, thymectomy (surgical removal of the thymus) of C57BL/6 mice during neonatal life (postnatal week 3–4) or adulthood (postnatal week 13·5) does not alter VAT Treg accumulation,^37^ suggesting that the VAT Tregs do not require constant replenishment from the thymus, at least beyond 3 weeks of life. A similar conclusion was drawn from Foxp3^DTR^ mice, which harbour a diphtheria toxin receptor (DTR) transgene ahead of the Foxp3 locus.^40^ The administration of diphtheria toxin permits systemic and specific deletion of all Foxp3‐expressing cells. Systemic Treg depletion at 8 or 13 weeks of age results in a failure of reconstructing the VAT Treg compartment, without influencing splenic Tregs.^37^ Yet, Treg ablation before 4 weeks yields no change in Tregs within VAT or spleen.^37^ Hence, VAT Tregs are probably seeded during the first 3 weeks of life. Once seeded, VAT Tregs may undergo clonal expansion, as suggested by the highly repetitive TCR sequencing results, and probably replenishes and maintains the VAT Treg compartment.^37^

### VAT Tregs suppress tissue inflammation and maintain energy homeostasis

The histopathology of obesity is defined by chronic low‐grade adipose tissue inflammation. Obesity is closely associated with the inability to respond to insulin, termed insulin resistance. In its severe form, obesity can further manifest to the pathology of type 2 diabetes, including elevated glucose and insulin in fasting individuals, lower insulin‐receptor activity, delayed blood glucose clearance (a poor glucose‐tolerance response), and increased glucose–insulin by‐products, as measured by the homeostatic model assessment of insulin resistance (reviewed in ref. 41, 42).

Obesity is associated with macrophage and CD8^+^ T‐cell accumulation in VAT, the production of pro‐inflammatory cytokines (TNF‐*α* and IL‐6), and a significant reduction of VAT Tregs.^21^, ^35^, ^43^, ^44^, ^45^ A meta‐analysis of 91 human clinical studies has revealed that type 2 diabetes is associated with increased IL‐6 and TNF‐*α*, and reduced Treg abundance in human peripheral blood.^46^ Indeed, murine VAT Tregs can suppress adipose tissue inflammation and potentially reduce diabetic pathology. Based on *in vitro* suppression assays, isolated murine VAT Tregs are immunosuppressive, and are not functionally different from splenic Tregs.^21^ In healthy lean mice, VAT Tregs also express high levels of the anti‐inflammatory cytokine IL‐10,^21^, ^35^, ^47^ further supporting their role as immunoregulators. Indeed, a number of *in vivo* studies describe VAT Treg suppression of other resident immune cells. First, Treg ablation leads to the induction of inflammatory mediators TNF‐*α*, IL‐6, RANTES and serum amyloid A‐3 within VAT.^21^ Similarly, anti‐CD25‐mediated Treg depletion in diabetic leptin‐deficient *db/db* mice elevates the pro‐inflammatory cytokine transcripts, *Ifng*, *Il6* and *Tnfa*, and down‐regulates the VAT Treg signature markers *Gata3*,* Ccr2*,* Klrg1* and *Cd69*.^48^ The loss of Tregs also results in pro‐inflammatory macrophage and monocyte accumulation, without affecting anti‐inflammatory monocytes, CD8^+^ T cells or B cells.^35^ In contrast, oral administration of anti‐CD3 antibody and *β*‐glucosylceramide in *ob/ob* mice augments adipose‐resident Tregs, while reducing CD11b^+^ F4/80^+^ macrophage abundance and *Tnfα* transcript in adipose tissue, subsequently dampening inflammation.^49^ These results provide clear evidence that VAT Tregs are essential to suppress adipose tissue inflammation.

Systemic Treg depletion in 10‐week‐old male Foxp3^DTR^ mice also leads to elevated fasting insulin levels, accompanied by a reduction in epididymal fat and liver insulin‐receptor activity.^21^ Similarly, Treg depletion in 6‐week‐old diabetic leptin‐deficient *db/db* mice also increases fasting glucose levels, lowers insulin sensitivity and elevates homeostatic model assessment of insulin resistance, relative to untreated *db/db* mice.^48^ In contrast, Treg augmentation through IL‐2–IL‐2 antibody complexes or adoptive transfer of lymphoid Tregs improves glucose tolerance,^21^, ^48^ suggesting a protective role for VAT Tregs in insulin resistance.

The administration of pioglitazone, an insulin‐sensitizing PPAR*γ* agonist, significantly increases VAT Treg numbers in high‐fat diet‐induced obese mice, and also lowers circulating glucose levels.^35^ Indeed, metabolic improvement of pioglitazone is diminished in obese Foxp3^cre^ PPAR*γ* ^fl/fl^ mice,^35^ indicating that PPAR*γ* is a critical regulator. It remains to be confirmed whether pioglitazone treatment alone in obese mice is enough to restore VAT Treg abundance and aggravate obesity‐induced insulin resistance to a homeostatic level. This view has been challenged by Bapat *et al*.,^36^ where specific loss of VAT Tregs in Foxp3^cre^ PPAR*γ* ^fl/fl^ mice does not impact glucose metabolism in lean, young (12‐week‐old) or high‐fat diet‐induced obese (24‐week‐old) mice. Instead, specific loss of VAT Tregs leads to improved fasting glucose and insulin levels in aged (36‐week‐old) mice,^36^ suggesting an opposing role for VAT Tregs in age‐associated insulin resistance. Nonetheless, there is an indisputable correlation between VAT Treg abundance and insulin sensitivity, and their essential role in maintaining metabolic homeostasis.

Despite preferential amphiregulin (Areg) expression in VAT Tregs,^21^, ^35^, ^43^, ^47^ this pathway remains to be explored in detail. Areg, an epidermal growth factor‐like molecule, is classified as a type‐2 associated cytokine, and has been associated with suppression of local inflammation and promotion of tissue repair (reviewed in ref. 50). The overexpression of Areg in obese *db/db* mice results in reduced perirenal and epididymal fat, and increased glucose and lipid metabolism‐related genes (including *Pparγ coactivator 1α* and *Tnfa*), implying that Areg is crucial for adipose tissue development.^51^ Whether this directly translates to the function(s) of VAT Treg‐derived Areg remains an open question, as Areg is also expressed by other immune cells, including mast cells, basophils, eosinophils and type 2 innate lymphoid cells.^50^

## Intestinal Tregs

Within the small and large intestines, the majority of immune cells reside within the mucosa, the innermost layer of the intestinal wall (reviewed in ref. 52). The mucosal wall consists of an epithelial layer, lamina propria and a thin underlying muscle layer (known as muscularis mucosa).^53^ Both small intestinal and colonic Tregs localize within the lamina propria of the intestinal mucosal wall,^54^, ^55^ contributing to ~35% and ~25% of residing CD4^+^ T cells, respectively.^56^ However, Treg are most abundant in the colon, and lowest in the duodenum of the small intestine.^57^

Similar to VAT, intestinal Tregs also adapt to their tissue environment. Of which, *Rorc*, a key Th17 lineage regulator encoding retinoic acid receptor‐related orphan receptor *γ*t (RoR*γ*t), is preferentially expressed in colonic Tregs, but not in other non‐lymphoid tissue or splenic Tregs.^58^ Of total colonic Tregs, around 40% are ROR*γ*t^+^ Helios^−^ and 30% are GATA3^+^, relative to >10% of Tregs in Peyer’s patches (intestinal lymphoid follicles) or in mesenteric LNs.^59^, ^60^ While ~30% of small intestinal Tregs express GATA3, only 15% are ROR*γ*t^+^ Helios^−^.^59^, ^60^ Notably, in both colonic and small intestinal Tregs, GATA3 and ROR*γ*t expression are mutually exclusive.^59^, ^60^ Overall, intestinal Tregs can be separated into three phenotypically distinct populations: GATA3^+^ Helios^+^, ROR*γ*t^+^ Helios^−^ and ROR*γ*t^−^ Helios^−^.

### Mixed origins of intestinal Tregs

Given their phenotypic heterogeneity, intestinal Tregs appear to be of mixed origin. In specific pathogen‐free (SPF) mice, 70% of colonic Tregs are negative for Helios,^56^ and 45% express low levels of *Nrp‐1*, in contrast to 11–19% of splenic or lymphoid Tregs,^61^ suggesting a pTreg origin. Contrary to the stable high expression in splenic Tregs, the percentage of Helios^hi^ or Nrp‐1^hi^ colonic Tregs declines from 70% to 80% at 1 week of age to ~30% at 8–10 weeks, implying an increasing contribution of pTregs.^62^ Although it is largely accepted that pTregs contribute to the establishment of intestinal Tregs, the majority of CDR3 TCR sequences of both colonic and small intestinal Tregs closely resemble that of thymic and peripheral LN Tregs.^63^ This repertoire was largely distinct from intestinal Teffs in transgenic mice with limited TCR repertoires,^63^ suggesting a tTreg origin. Minimal tTreg expansion was also observed when transferring *Foxp3* ^gfp^ *Rag1* ^−/−^ thymocytes retrovirally transfected with colonic Treg TCRs, into *Rag1* ^−/−^ recipients (that lack T and B cells).^64^ Furthermore, transferring LN Teffs to Treg‐depleted mice, Teffs can acquire a ROR*γ*t^+^ Helios^−^ and Helios^+^ colonic Treg phenotype, depending on the level of nerve growth factor IB (otherwise known as Nur77).^65^ Nur77 functions as an important regulator of cell survival, inflammation, but also as an early reporter for antigen‐specific signalling.^66^ Taken together, both tTregs and pTregs contribute to the establishment of colonic Tregs.

Colonic Tregs are highly responsive towards microbiome colonization. The microbiome refers to the microorganisms (bacteria, archaea, protozoa, fungi or viruses) sharing a commensal, symbiotic or pathogenic relationship with their host of residence. Increasing evidence illustrates their vital role in regulating host metabolism, drug utilization, maintaining homeostasis, and ultimately host health (reviewed in ref. 67). In germ‐free C57BL/6 mice, only 10% of colonic CD4^+^ T cells are Tregs, in contrast to ~35% in SPF C57BL/6.^56^ Treatment with Gram‐positive antibiotics (vancomycin) in SPF mice leads to an ~10% reduction in colonic Tregs, whereas no change was observed in SPF mice treated with Gram‐negative antibiotics (polymyxin B), indicating that Gram‐positive bacteria may promote colonic Treg establishment.^56^ Using *in vitro* co‐cultivation of colonic Tregs and dendritic cells primed with autoclaved colonic contents, colonic Treg TCRs appear to engage microbiome‐derived antigens.^64^ Unsurprisingly, colonic Treg activation during colonization is dependent on Toll‐like receptor signalling. Co‐deficiency of the Toll‐like receptor adaptor molecules (MyD88 and Ticam‐1) in germ‐free mice ablates colonic Treg accumulation during microbial colonization.^68^ Conversely, Tregs residing in small intestinal lamina propria are unaffected by microbiome diversity, instead their induction appears to be largely driven by dietary antigens.^69^ Hence, intestinal Treg establishment is probably facilitated by the environmental antigens present within organs, with microbiota as a key component.

### Colonic Tregs suppress ongoing intestinal inflammation

Compromising colonic Treg function results in unresolved and ongoing inflammation, but their contribution to short‐term homeostatic maintenance appears less important. Deletion of Tregs for a period of 10 days in *Foxp3^DTR^* mice does not impact lymphocytic infiltration or proliferation in the colon.^70^ Yet, IL‐10 receptor deficiency in Tregs (*Foxp3^cre^ Il10ra^fl/fl^*) manifests in severe colitis in aged mice (18–20 weeks), but not younger mice (8–10 weeks).^71^ Similarly, patients with single point mutations in CTLA4 are associated with extensive gastrointestinal inflammation, indicating a major functional role for Tregs in controlling long‐term colon pathology.^72^, ^73^

Transforming growth factor‐*β* signalling is a key component in regulating T‐cell proliferation and activation. For example, TGF*β*_1_ deficiency in CD4^+^ T cells or in OX40^+^ T cells leads to severe colitis, accompanied by elevated IFN‐*γ*.^74^, ^75^ Yet, during steady‐state, deficiency of TGF‐*β*_1_ or its receptor (TGF‐*β*R) in Tregs, does not impact IFN‐*γ* or IL‐17 production in small intestinal or colonic intraepithelial lymphocytes.^75^, ^76^ Similarly, colonic inflammation is absent in mice harbouring Treg conditional deletion of the TGF‐*β* activator (integrin *α*_v_ *β*_8_, Itgb8).^77^ Hence, Treg‐derived TGF‐*β* signalling contributes minimally to the regulation of steady‐state colonic homeostasis.

Colitis can be artificially induced by transferring naive CD45RB^high^ CD4^+^ T cells into immunodeficient Rag1^−/−^ mice, characterized by colon thickening, and ultimately weight loss.^78^ Co‐transfer of CD4^+^ CD25^+^ Tregs is protective against CD4^+^ Teff cell‐mediated inflammation.^78^ However, co‐transferring TGF‐*β*_1_‐deficient splenic Tregs induces an IFN‐*γ*‐mediated response and failure to resolve colitis.^79^ Similarly, co‐transferring TGF‐*β*RI‐deficient Tregs leads to an increase in monocyte and neutrophil infiltration in the colon.^76^ Likewise, a more severe colitis is observed when transferring Itgb8‐negative Tregs into inflamed Rag2^−/−^ recipients. This exacerbated pathology was attributed to increased neutrophils, monocyte/macrophages, IFN‐*γ*‐expressing and IL‐17‐expressing CD4^+^ T cells within the colon.^77^ Importantly, TGF‐*β*RI‐deficient Tregs fail to accumulate or be retained within Rag1^−/−^ colon, explaining the uncontrolled inflammatory response.^76^ In dextran sulphate sodium‐mediated colitis, the lack of Itgb8^+^ Tregs also exacerbates pathology, including the elevation of CD4^+^ T cells without altering Treg migration, activation or stability.^77^ Taken together, colonic Tregs regulate ongoing inflammation via TGF‐*β* signalling, and may contribute minimally during the steady state.

Distinct intestinal Treg subpopulations appear to possess specialized functional roles in the suppression of ongoing inflammation. GATA3^+^ Helios^+^ Tregs function as major immunosuppressors during intestinal inflammation. Mice harbouring Gata3‐deficient Tregs (Foxp3^IRES‐Cre^ Gata3^fl/fl^) do not display an inflammatory phenotype, nor changes in Treg abundance.^60^ Yet, upon injury, colonic GATA3^+^ Helios^+^ Tregs express increased levels of IL‐10 and TGF‐*β* in an IL‐33‐dependent manner.^80^ Co‐transfer of Gata3‐negative Tregs from *OX40^cre^ Gata3^fl/fl^*, or *Foxp3^EGFP‐cre^ Gata‐3^fl/fl^* mice, with naive T cells into *Rag1^−/−^* recipients induces severe colitis and weight loss,^60^, ^81^ suggesting that Tregs use Gata3 to limit tissue inflammation. The role of Gata3 was further confirmed in a competitive bone marrow chimera model, where CD45.2^+^ Gata3‐deficient and CD45.1^+^ CD45.2^+^ wild‐type cells were transferred into lethally irradiated CD45.1^+^ recipients.^60^ While Teff accumulation is unaffected, Gata3 deficiency hinders Treg infiltration to inflamed intestine.^60^ This was also true for Tregs within the spleen and mesenteric LNs.^60^ Importantly, intestinal Gata3‐deficient Tregs express significantly higher levels of ROR*γ*t and IL‐17A relative to wild‐type cells,^60^ indicating that Gata3 may also function as a restrictor of Th17 immunity. However, Gata3‐negative splenic Tregs also express higher *Rorc* and *IL‐17a* transcript. Although Gata3 is critical in facilitating intestinal Treg function, its regulatory role in suppressing Th17 immunity is unlikely to be intestinal Treg‐specific.

Ablation of ROR*γ*t^+^ colonic Tregs results in an up‐regulation of Teff‐derived IL‐17*α* and IFN‐*γ*, and the development of severe colitis.^59^ ROR*γ*t^+^ Tregs also induce tolerance towards the pathogenic microbe *Helicobacter hepaticus*, via the cMaf transcription factor, which promotes IL‐10 secretion and restricts Th17 polarization.^82^ However, it remains controversial as to what level human ROR*γ*t^+^ Tregs control intestinal inflammation. On the one hand, the abundance of human ROR*γ*t^+^ Tregs is comparable between healthy individuals and patients with inflammatory bowel disease.^59^ Yet, more human ROR*γ*t^+^ Tregs were detected in dysplastic than non‐dysplastic histology from individuals with ulcerative colitis.^83^

### Intestinal Tregs facilitate tissue repair

Amphiregulin is also highly expressed in intestinal Tregs.^58^ However, mutants lacking Areg‐expressing Tregs show no gross phenotypic pathology, nor changes in the frequency of colon‐resident Th1, Th2 or Th17 cells.^84^ Although Treg‐derived Areg appears dispensable, Areg derived from other cell types may impact Treg control of colonic inflammation. Treg‐mediated suppression is less effective in Areg‐deficient Rag1^−/−^ (Areg^−/−^ Rag1^−/−^) mice relative to Rag1^−/−^ controls.^85^ In fact, sorted CD25^+^ Tregs from CD4^cre^ Egfr^fl/fl^ mice, which lack the Areg receptor epidermal growth factor receptor (EGFR), failed to suppress disease development,^85^ indicating the importance of Areg in enhancing Treg immunosuppression. Additionally, Areg^−/−^ mutants have significantly impaired intestinal epithelial regeneration after radiation exposure.^86^ Similarly, tissue Tregs have also been associated with the maintenance of intestinal stem cells.^87^ Whether Treg‐derived Areg is required to facilitate tissue repair remains currently unknown.

## Skin Tregs

Both human and mouse skin are composed of epidermis and dermis, with hair follicles (HFs) interspersed between their junctions (reviewed in ref. 88). The majority of murine Tregs localize near HFs.^89^ Similarly, the abundance of human Tregs is closely associated with hair density, with more skin Tregs in areas of high hair density (such as scalp or face).^90^ In the steady state, skin Tregs contribute to an average of 50% of adult skin‐resident CD4^+^ T cells in mice,^91^ and 20% in humans.^90^ Of murine neonatal CD4^+^ cells, >80% are murine Tregs that express high levels of CTLA4 and ICOS, but their numbers are reduced to 50% in adulthood.^91^ Murine skin Tregs are transcriptomically similar to colonic Tregs, but they uniquely express genes such as *Dgat2* (related to lipid synthesis in skin).^24^ Bulk RNA sequencing of dorsal skin and LN Tregs revealed preferential expression of Jagged1 (Jag1), a Notch signalling ligand, in skin Tregs.^92^ In humans, the mitochondrial protein arginase 2 (*Arg2*) is also preferentially expressed in skin Tregs, relative to skin Teffs or circulating Tregs.^93^ Using CRISPR‐Cas9‐mediated ARG2 deletion, the authors concluded that Tregs use this pathway to maintain a tissue‐specific signature.^93^

### Origin and residency of skin Tregs

Skin Tregs accumulate during a specific neonatal period (postnatal days 6–13) and are thought to originate from the thymus.^91^ During this period, blockade of thymic or LN T‐cell egress, by the administration of sphingosine‐1‐phosphate receptor antagonist FTY720, results in ~10‐fold reduction in skin Treg numbers. This is also associated with an increase in thymic Tregs,^91^ indicating that skin Tregs are potentially thymus‐derived. Until now, a comprehensive CDR3 sequencing comparing skin Tregs, skin Teffs and their counterparts in spleen or lymph nodes has not been performed. Hence, skin Treg origin remains inconclusive.

Further evidence comes from Kaede transgenic mice that harbour a photoactivatable fluorescence protein. The Kaede protein undergoes irreversible conversion from green to red fluorescence upon violet light exposure, allowing non‐invasive *in vivo* tracing of cell migration.^94^ In inflamed ear skin, ~30% of Tregs have permanent residency, whereas 50% are migratory.^95^ These migratory Tregs express higher levels of CTLA4 and Nrp1, but lower levels of CD25 and CD39 than those remaining in skin.^95^ Given the increased expression of Nrp1, these migratory Tregs are probably of thymic origin. Whether this finding can extend our understanding of dorsal skin Treg migration during homeostasis remain unknown. In addition, Treg expression of FuT7, an enzyme facilitating E‐selectin and P‐selectin binding, is required for optimal Treg trafficking to both inflamed and non‐inflamed skin,^96^ whereas their retention appears to require IL‐7, but not IL‐2.^97^

Similar to the colon, the skin harbours a large quantity and diversity of microbial species. In germ‐free mice, there is a 20% reduction in neonatal skin Tregs,^98^ but no differences are observed in adults.^99^ Conversely, other T‐cell populations (such as CD4^+^ Foxp3^−^ conventional T cells, CD8^+^ T cells, dermal *γδ* or dermal epithelial T cells) remain unaffected in neonatal skin,^98^ indicating that the microbiome preferentially orchestrates skin Treg accumulation. The microbiome drives the production of hair follicle‐derived CCL20, the ligand for the skin‐homing receptor CCR6, and subsequently facilitates skin Treg migration into neonatal skin.^98^ Successful accumulation of skin Tregs to neonatal skin, relative to adulthood, appears critical for the establishment of microbiome tolerance.^91^ However, only specific strains can induce skin Treg tolerance. For example, neonatal colonization with the commensal *Staphylococcus epidermidis*, but not with the pathobiont *Staphylococcus aureus*, limits inflammation upon repeated encounter during adulthood.^100^ The discrimination between commensals and pathobionts is attributed to the *S*.* aureus*‐derived *α*‐toxin and its regulation of IL‐1*β* production in skin.^100^ Taken together, the timing of tissue Treg establishment and the nature of the microbiome plays a major role in instructing Treg‐mediated tolerance in skin.

### Skin Tregs facilitate wound healing and control fibroblast activation

Similar to the colon, short‐term skin Treg deletion does not appear to impact immune homeostasis. Overt signs of cutaneous inflammation are absent during periods of acute Treg depletion in Foxp3^DTR^ mice. These include epidermal hyperplasia, the abundance of other skin‐resident immune cells, and the production of the pro‐inflammatory cytokines IFN‐*γ*, TNF‐*α* and IL‐17.^92^, ^101^ However, Treg depletion up‐regulates the Th2 cytokines IL‐13 and IL‐4.^101^ At the single‐cell level, *Gata3* transcript is most abundant in murine skin Tregs, relative to other Th lineage transcription factors *Rorc* and *Tbx21*, suggesting that skin Tregs may be skewed towards Th2 regulation.^101^ Conditional deletion of GATA3 in Tregs (*Foxp3* ^YFP‐Cre^ *Gata3* ^fl/fl^) results in Type 2 associated skin inflammation, accompanied by a reduction in Treg abundance and activation. Treg‐specific deletion of GATA3 also manifests in polymorphonuclear cell infiltrate, as well as IL‐5 and IL‐13 producing CD8^+^ T cells in skin.^101^, ^102^ These findings imply that skin Tregs (or at least a subset thereof) maintain skin homeostasis via regulation of Th2 immunity.

In full‐thickness wounding of mouse dorsal skin, Treg numbers increase approximately 20‐fold, which is concurrent with increased expression of activation markers (*Ctla4*,* Icos* and *Cd25*) at days 3 and 7 post‐injury.^103^ Consequentially, Treg ablation delays cutaneous wound closure, and is associated with the accumulation of IFN‐*γ*‐producing T cells and CD11b^+^ Ly‐6C^high^ pro‐inflammatory macrophage in wounded skin,^103^ demonstrating that Tregs are indispensable for resolving skin inflammation. Delayed wound closure was observed upon Treg‐specific deletion of the Areg receptor, EGFR, indicating a requirement for locally available Areg to sustain the kinetics of tissue repair.^103^ Similarly, skin Tregs also facilitate repair of the injured epithelial barrier by suppression of IL‐17A, CXCL5, and subsequent neutrophil accumulation.^104^ In turn, Tregs promote hair follicle stem cell (HFSC) activation, driving epidermal‐differentiation genes (such as *filaggrin*, *keratin1*) and restoration of the epidermal barrier.^104^ Overall, skin Tregs promote cutaneous repair by facilitating early innate immunity.

Skin Tregs also play a major role in fibrotic disease. Using *α*SMA‐RFP/Foxp3^DTR^ and female heterozygous Foxp3^DTR^ mice, both acute (5 days) and chronic (4 weeks) steady‐state Treg depletion resulted in the accumulation of profibrogenic myofibroblasts, and up‐regulation of profibrotic genes (*Col3a1*,* αSMA* and *Hsp47*).^101^ Chronic depletion also down‐regulates IL‐10 production and anti‐fibrosis genes (*Mmp2a*,* Mmp8* and *Bmp7*), with increased dermal collagen density and dermal thickness, culminating in a fibrotic appearing pathology.^101^ Severe fibrotic pathology was observed upon partial depletion of Tregs in *α*SMA‐RFP/ Foxp3^DTR+/−^ treated with the dermal fibrosis‐inducing agent, bleomycin.^101^ Hence, skin Tregs appear indispensable for suppressing or reducing steady‐state and injury‐induced fibrosis. These findings were largely recapitulated in bleomycin‐treated Foxp3^creERT2^ Gata3^fl/fl^ mice, revealing Gata3^+^ Tregs as a core subset contributing to the control of Th2‐mediated skin fibrosis.^101^

Interestingly, during both the acute and chronic Treg depletion models, TGF‐β transcripts (such as*Tgfbr1*, *Tgfb3* and *Smad2)* in dorsal skin are up‐regulated.^101^ It is well‐established that TGF‐*β* signalling plays a critical role in maintaining the cutaneous barrier, including re‐epithelialization and the retention of memory T cells or Langerhans cells in skin (reviewed in ref. 105). In delayed‐type II hypersensitivity, integrin *α*_v_ *β*_8_ (Itgb8) in Foxp3^+^ Tregs is required to control ear skin inflammation and IFN‐*γ*‐producing CD4^+^ and CD8^+^ T cells.^77^ However, during the steady state, cutaneous pathology was absent in mice lacking TGF‐*β*RI in Tregs.^76^ Taken together, similar to intestinal Tregs, TGF‐*β* signalling also plays a direct role in skin Treg‐mediated suppression of ongoing inflammation. It remains unclear whether Treg‐derived TGF‐*β* impacts other immune populations, such as memory T cells and dendritic cells in skin.

### Skin Tregs facilitate hair regeneration

In murine skin, HFs cycle between perpetual bouts of growth arrest (telogen) and activation (anagen) to form new hair shafts. This process is mediated by epithelial stem cells within the HF bulge region (HFSCs).^106^ Depletion of Tregs during telogen significantly impairs anagen induction as evidenced by HFSC proliferation and differentiation defects, consequently impacting hair regeneration.^92^ Yet, co‐depletion of Tregs with other immune effector cells, or neutralization of the IFN‐*γ* pathway, is unable to rescue HFSC activation.^92^ This in turn suggests that skin Tregs may regulate HF regeneration outside their conventional role in suppressing skin inflammation. It was later revealed that the Notch ligand Jagged1 is preferentially expressed in skin Tregs, relative to skin‐draining LN Tregs.^92^ Conditional deletion of Jag1 in Tregs, using Foxp3^YFP‐cre^ Jag1^fl/fl^ mutants, significantly impairs bulge HFSC proliferation, HFSC differentiation transcripts and, subsequently, anagen induction.^92^ However, the mechanism(s) underlying the induction and maintenance of Jag1^+^ Tregs, and whether skin Tregs use the Notch signalling pathway to directly mediate HFSC function, remains unknown.

## Skeletal muscle Tregs

Skeletal muscle is composed of multiple myofibres and connective tissues. The ability to activate quiescent satellite cells (muscle stem cells) on demand contributes to the remarkable capacity of skeletal muscle regeneration (reviewed in ref. 107). Unlike the previously discussed tissues, steady‐state skeletal muscle harbours a minimal quantity of Tregs, accounting for only 10% of CD4^+^ T cells, less than their counterparts in spleen.^58^, ^108^ Instead, Tregs are analysed in injured or inflamed muscle. Cardiotoxin‐induced muscle injury triggers rapid Treg accumulation, accounting for 40%–50% of CD4^+^ T cells at day 4 post‐injury.^58^, ^108^ Similarly, in both humans and experimental mouse models of Duchenne muscular dystrophy, muscle Treg numbers are significantly elevated.^109^ Unsurprisingly, this is not exclusive to muscle Tregs, as the number of muscle Teffs share a similar pattern.^58^, ^108^, ^109^

### Unclear origin of injured muscle Tregs

The origin of Tregs in injured muscle appears complex to interpret. Although the number of muscle Tregs declines drastically after 1 week post‐injury, it remains eightfold higher than uninjured muscle.^58^, ^108^ There are potentially two possible origins of elevated Treg populations in injured muscle: (i) they represent infiltrating Tregs, which originate from other lymphatic organs, or (ii) they are derived from the expansion of a rare muscle‐resident Treg population. Upon successful tissue repair, Tregs migrate out of or do not survive in muscle. Experimental evidence for and against these ideas so far is conflicting. Administration of the T‐cell egress inhibitor FTY720, before cardiotoxin‐induced muscle injury, lowers Treg numbers, but not their percentage among CD4^+^ cells, nor total CD45^+^ cell abundance.^108^ This indicates that the accumulation of muscle Tregs are likely dependent on T‐cell recruitment from other lymphatic organs. Yet, through *in vivo* tracing in Kaede mice, only a small fraction of Tregs in injured muscle traffic from cervical lymph nodes.^108^ Although this does not exclude the possibility that muscle Tregs may be recruited from other lymphatic organs, at least a portion of muscle Tregs infiltrate upon injury. Furthermore, 84% of post‐injury muscle Tregs are highly proliferative, expressing higher levels of Ki67 and EdU relative to splenic Tregs or muscle/splenic Teffs.^58^, ^109^ Based on CDR3 sequencing of TCR‐*α* and TCR‐*β* chains, 20%–40% of muscle Tregs share the same TCR sequences, suggesting clonal expansion.^58^ Importantly, the TCR sequences of muscle Tregs are distinct from muscle Teffs,^58^ demonstrating muscle Tregs are unlikely to originate from pTregs. In a mutant strain with fixed TCR‐*α* and TCR‐*β*, TCR specificity drives muscle Treg phenotypic adaptation and accumulation in cardiotoxin‐injured muscle.^110^ Hence, muscle Treg accumulation may be derived from the local proliferation of existing Tregs as well. However, the specific fraction in which existing muscle and infiltrating Tregs contribute to the accumulation of Tregs in injured muscle remains to be fully elucidated.

### Muscle Tregs promote muscle repair

Under homeostasis, Tregs play a major role in facilitating macrophage transition from a pro‐inflammatory (CD11b^+^ Ly6c^hi^) to anti‐inflammatory (CD11b^+^ Ly6c^low^) phenotype (reviewed in ref. 29). This transition is absent when Tregs are depleted post‐cardiotoxin injury.^58^ Unsurprisingly, this is only observed in injured muscle, but not uninjured muscle,^58^ suggesting that the functions of muscle Tregs are unlikely to be the same during the steady state, if any. Furthermore, muscle Tregs are also required to limit pro‐inflammatory IFN‐*γ* production, and the MHCII^+^ macrophage response.^109^, ^111^ This is accompanied by increased fibrosis, but also a reduction in regenerative nucleated myofibres, and attenuated expression of genes related to muscle repair (*Mmp12*,* C1qa*,* Myog*), immune response/inflammation (*Cd8a*,* Ccl17*,* Arg1*) and matrix protein (*Col6a5*),^58^ suggesting a failure in muscle regeneration. Taken together, muscle Tregs, at least upon cardiotoxin‐induced injury, facilitate the shift of macrophage phenotype, suppressing fibrosis and mediating muscle repair.

The contribution of muscle Tregs to tissue repair appears to be Areg‐mediated. The administration of recombinant Areg in cardiotoxin‐injured Treg‐depleted mice up‐regulates transcripts associated with muscle function (*Myl2*,* Myl3* and *Me1*), and down‐regulates muscle development/differentiation (*Adam12* and *Myog*) and fibrosis‐related transcripts (*Col1a1*, *Col2a1*, *Col3a1* and *Col4a1*).^58^ Hence, Areg may maintain satellite cell ‘stemness’ and fibrosis suppression, at least at the molecular level. *In vitro* culture of wild‐type satellite cells with Areg enhances colony‐forming efficiency and myogenic differentiation,^58^ suggesting that Areg can directly impact muscle stem cells. However, this remains to be shown *in vivo*.

Similarly, infection with the protozoan parasite *Toxoplasma gondii* augments muscle Treg abundance and Areg production, with subsequent loss of tissue function.^112^ These findings indicate the induction of muscle Tregs, as well as Treg‐derived Areg, are probably regulated by muscle injury, irrespective of the nature of injury. However, in this model, Treg depletion did not impact the absolute number of pro‐ and anti‐inflammatory macrophages.^112^ Instead, there is an increase in centrally nucleated muscle fibres. In summary, it appears that Tregs hinder, rather than promote, muscle regeneration upon *T*.* gondii* infection.^112^ Of note, in fibrosis‐resistant ADAM17 PTC knockout mice, administration of recombinant Areg induces kidney fibrosis in response to post‐ischaemia reperfusion injury.^113^ These studies collectively imply that the role(s) played by muscle Tregs may be dependent on the injury stimuli.

Duchenne muscular dystrophy pathogenesis is largely characterized by chronic muscle inflammation and eventual loss of structural tissue integrity.^58^, ^109^ Relative to control muscle, Treg numbers (irrespective of anatomical location), their activation and IL‐10 production, are elevated in diseased muscle.^58^, ^109^ Anti‐CD25‐mediated Treg depletion in an experimental model leads to increased IFN‐*γ* expression, without influencing IL‐10 expression. The absence of Tregs also down‐regulates genes implicated in muscle homeostasis, but up‐regulates muscle‐reparative and fibrosis‐promoting genes, such as osteopontin (*Spp1*) and connective‐tissue growth factor (*Ctgf)*.^58^ Conversely, augmentation of muscle Tregs through IL‐2–IL‐2 complexes attenuates muscle damage.^58^ Taken together, muscle Tregs regulate muscle fibro‐pathology and regeneration in response to both acute and chronic injury.

## Future directions

**Figure 1:**
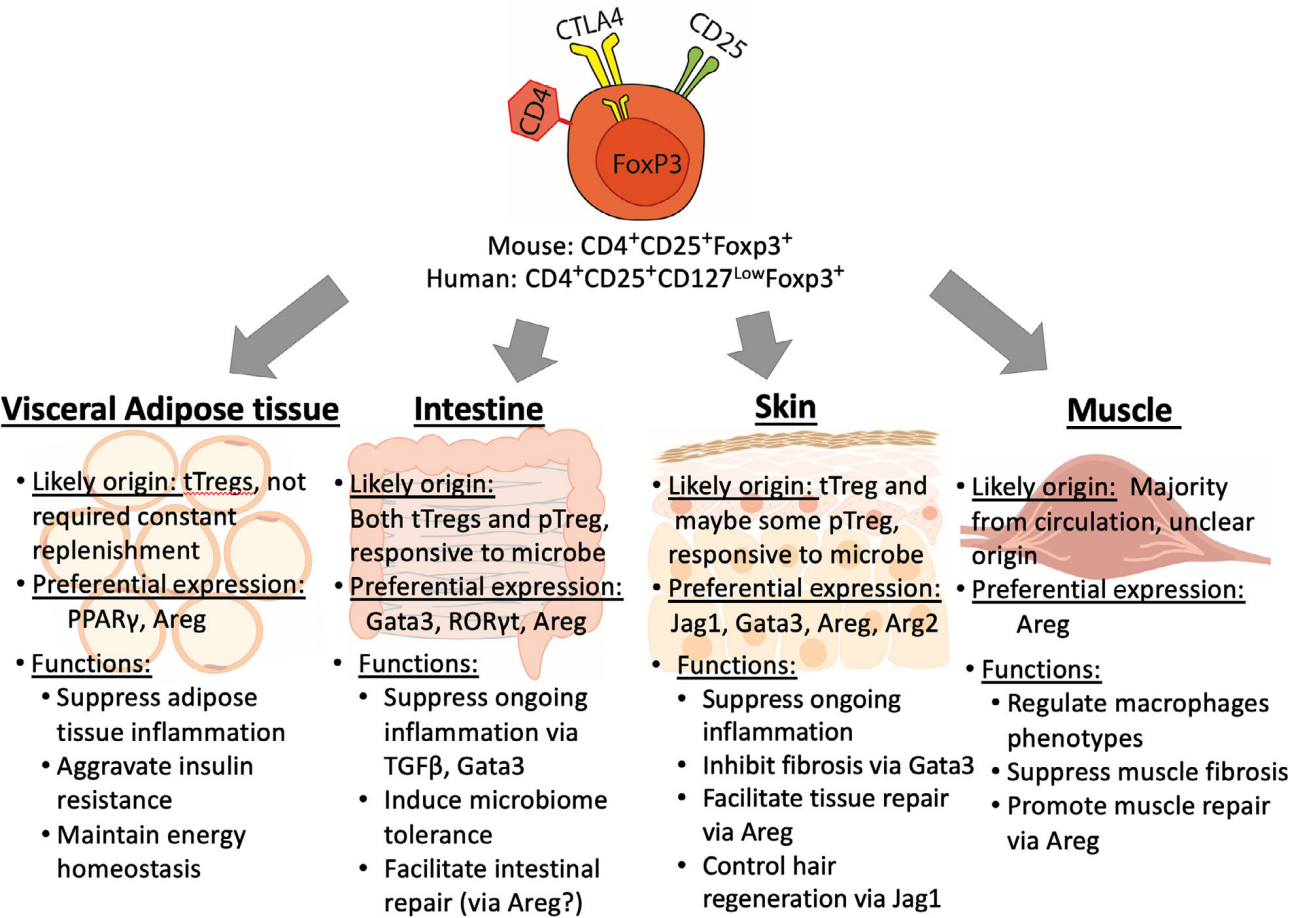
Regulatory T cells (Tregs) residing within four major tissue sites. The majority of visceral adipose tissue Tregs, marked by their preferential expression of peroxisome proliferator‐activated receptor *γ* (PPAR*γ*), are mainly thymically derived. Contrarily, intestinal Tregs are highly heterogeneous, in both preferential expression and origin. Some evidence has suggested skin Tregs are of thymic origin, and accumulate during neonatal life. Uniquely, the majority of muscle Tregs are derived from the circulation, but retain a preferential transcriptome relative to lymphoid Tregs. These four tissue Treg populations serve as important regulators of tissue inflammation and fibrosis, as well as playing a major role in facilitating tissue repair.

In summary, we have presented the current advancements in the phenotypic diversity and functions of tissue Tregs (Fig. 1). However, many areas remain underexplored. Below, we highlight the most pertinent.

### A potential Areg–TGF‐*β* axis in facilitating Treg‐mediated tissue repair

In this review we have focused on Tregs in four organs with the most experimental data, but it is important to note that Tregs also reside in other tissues, and equally play a central role in maintaining homeostasis. For example, Treg depletion can aggravate lung fibrosis in *Aspergillus* fungus‐exposed mice, accompanied by an increased infiltration of inflammatory cells, in particular CD103^lo^ GATA3^hi^ T cells.^114^ During PR8‐OTI influenza virus infection, Treg‐derived Areg preserves lung tissue integrity and blood oxygen saturation.^84^ Similarly, after ischaemic stroke, Treg‐derived Areg was required to regulate neurotoxic astrogliosis .^115^ Despite the unanimous role of Areg in multiple tissue Treg populations, the exact molecular mechanisms of how Tregs use this pathway for tissue regeneration is not fully understood. One possible interconnection may be with the TGF‐*β* pathway. After acute lung and liver injury, macrophage‐derived Areg locally activates TGF‐*β* on mesenchymal stromal cells (pericytes), by induction of integrin‐*α*_v_ complexes.^116^ These pericytes then differentiate into collagen‐producing myofibroblasts, promoting re‐vascularization and tissue restoration.^116^ It is well‐established that TGF‐*β*, along with IL‐15 and T‐box transcription factors, plays a major role in the survival and development of tissue‐resident CD8^+^ CD103^+^ memory T cells,^117^ as well as Treg development and function.^74^ In addition, skin Treg abundance is significantly reduced in Foxp3^cre^ EGFR^fl/fl^ mice, suggesting a dependency on locally available Areg.^103^ As discussed, Treg‐derived Areg is critical for the control of intestinal and skin inflammation. Hence, it is plausible that Areg, in co‐operation with TGF‐*β*, may indirectly enable the self‐regulation of tissue Treg survival and function.

### Interconnection between Tregs at different tissue sites

An active and exciting area of investigation is whether the functions of Tregs residing in different tissues interconnect with each other. Emerging physiological and pathological evidence has repeatedly demonstrated an intimate, bidirectional association between skin and intestinal immune homeostasis (reviewed in ref. 118). Indeed, Treg subtypes are spatially localized in their unique niches but appear to be influenced by similar external cues. For instance, the repertoire and exposure to commensal and pathogenic microbiota is a major contributor to the regulation of both skin and colonic Treg abundance. More recently, it has been hypothesized that the gut microbiome can also influence skin Treg abundance and function(s), through the gut–skin axis. Several studies have suggested that oral consumption of specific microbial strains can influence cutaneous inflammation and wound healing processes (reviewed in ref. 118). For example, oral consumption of *Lactobacillus reuteri* accelerates healing of excision‐induced wounds in murine dorsal skin.^119^ Adoptive transfer of lymphoid Tregs from *L*.* reuteri*‐infected mice into Rag2^−/−^ recipients promotes the localization of Tregs proximal to skin wounded sites, resulting in accelerated healing.^119^ Contrarily, anti‐CD25‐mediated Treg depletion abolishes the improved response observed in the *L*.* reuteri*‐infected group,^119^ suggesting that *L*.* reuteri*‐mediated wound healing is facilitated by resident tissue Tregs, or a subset thereof. Similarly, oral administration of *Lactobacillus casei* enhances skin Treg abundance, IL‐10 production and delayed skin inflammation.^120^ The gut–skin axis remains to yet be fully explored, but it is an exciting field that may reveal a potential cross‐link between immunity in different organs, and whether this influences global tissue homeostasis.

### Current Treg immunotherapies

In recent years, increasing research has focused on the utilization of Tregs for immunotherapy. Traditionally, inflammation may be controlled by immunosuppressants (in the forms of drug metabolizing enzymes, hormone‐based or small molecules) in autoimmune diseases and for the prevention of transplantation rejection. One example is Azathioprine, which is commonly used to inhibit lymphocyte proliferation through inhibition of purine synthesis to delay graft rejection and as a treatment for rheumatoid arthritis or inflammatory bowel disease (reviewed in ref. 121). Besides the limited efficacy, adverse toxicity and undesirable secondary effects, the long‐term use of immunosuppressants can be detrimental overall.^122^, ^123^ There are currently three major Treg‐targeting approaches. (i) using known Treg co‐stimulatory pathways to suppress T‐cell activation. For example, CTLA4‐Ig (a fusion CTLA4 protein with human immunoglobulin) blocks the CD28/B7 co‐stimulation signal, and has shown high efficacy in rheumatoid arthritis and type 1 diabetes;^124^, ^125^ (ii) inducing *in vivo* expansion of Tregs and/or enhancing their functions with IL‐2, rapamycin or anti‐CD3. Low‐dose IL‐2 has been effective in chronic graft‐versus‐host disease to promote Treg proliferation and thymic export, rescue IL‐7 and IL‐15 levels, while showing a minimal effect on Teffs;^126^ and (iii) using adoptive transfer of *ex vivo* stimulated and expanded Tregs from patients’ peripheral blood mononuclear cells to ameliorate type I diabetes or delay solid organ transplant rejection. However, specificity remains a major obstacle in these therapies (reviewed in ref. 127, 128).

Early evidence of Tregs with defined antigen specificity (chimeric antigen receptor Tregs, CAR‐Tregs) appear promising. Although not clinically approved, multiple pre‐clinical studies have indicated the potential of CAR‐Tregs to induce immunological tolerance (reviewed in ref. 129). For example, transfer of HLA‐A2‐specific CAR‐Tregs can prevent HLA‐A2^+^ human skin graft rejection and promote graft survival in humanized mice.^130^, ^131^ One construct of CAR‐Tregs has proved beneficial over polyclonal Tregs, while limiting bystander cytotoxicity,^131^ suggesting that antigen specificity can improve therapeutic outcomes. A recent study has also shed light on the importance of Treg TCR specificity for muscle Treg accumulation, phenotype and function.^110^ Hence, elucidating the nature of antigens recognized by Tregs within tissue sites may be essential in designing therapies to induce immune tolerance.

However, CAR‐Treg therapies are limited by antigen selection, *in vivo* persistence and potential off‐target cytotoxicity (reviewed in ref. 129). Homing to target tissue is possibly the biggest hurdle. One possibility will be to engineer expression of tissue‐homing chemokine receptors (such as CCR6 for skin and CCR9 for intestine), creating an artificial means by which Tregs can infiltrate target tissues. It has been shown that Treg sequential migration to inflamed allografts helps to shape Treg phenotypes and immunosuppressive function within the tissue,^132^ proposing that Treg flux may be associated with their phenotypic diversity. Fully elucidating the tissue Treg phenotypes, and how this translates to each tissue Treg function(s) will be a critical piece of the puzzle in developing tissue‐specific CAR‐Treg therapy.

### Targeting tissue Tregs *in situ*

Furthermore, as highlighted in this review, immunosuppression is not the sole function of tissue Tregs. Conventional antigen recognition through the TCR may be one of many pathways to initiate tissue Treg‐mediated repair. In the lung, Treg‐mediated tissue repair functions in a TCR‐independent fashion and instead relies on IL‐18 or IL‐33 signalling.^84^ Interleukin‐33 is also required for the induction of IL‐13‐producing ST2^+^ Tregs during acute lung injury.^133^ In this scenario, rather than employing Tregs to suppress undesired target cells, a mechanism to activate Treg functions in a specific manner will be necessary. One potential method is the use of bi‐specific antibodies, which can bind to two unique antigens simultaneously. It may be possible to redirect the proximity between a given cell type with a second target cell within tissues. One clinically approved construct is blinatumomab, with anti‐CD3 targeting CD8^+^ T cells and anti‐CD19 against tumour cells. The link‐crossing between these two cell types ultimately leads to tumour eradication (reviewed in ref. 134). Hypothetically, one could engineer a bi‐specific antibody with a tissue ligand and Treg‐tissue‐specific expressed receptor to cross‐link Tregs with a parenchymal, or even stem cell. By targeting two receptors that are uniquely expressed in the tissue, there is potential to increase spatial co‐localization compared with monospecific antibodies. This in turn may reduce the risk of systemic Treg activation and improve efficacy.

## Concluding remarks

In summary, we have discussed the experimental evidence that Tregs residing in multiple tissue compartments share core phenotypic signatures and carry out immunosuppression. However, Tregs adapt to their respective environment, and express tissue‐specific factors that confer distinct roles in tissue repair. To clinically exploit these cells will require a more in‐depth understanding of the factors that regulate and maintain tissue Treg heterogeneity. Given that Tregs exert the majority of their functions in tissue sites, it is imperative the Treg community focuses on augmenting specific tissue‐resident subsets for the treatment of tissue regenerative and inflammatory disorders.

## Disclosures

The authors have no competing interests to declare.
